# Reviews

**Published:** 1884-06

**Authors:** 


### Book Reviews.

A PRACTICAL TREATISE ON MATERIA MEDICA AND THERAPEUTICS. By ROBERTS BATHOLOW, M.A., M.D., LLD. Professor of Materia Medica and General Therapeutics in the Jefferson Medical College of Philadelphia, etc. Fifth edition, revised and enlarged, 8vo. cloth, pp. xxii–738, New York: D. Appleton & Co., 1884.

This, as well as the work of the same author on Practice, is one of the many treatises of which the American medical profession feel proud, and it will pass to posterity as one of the medical classics, for it embodies in its tersely written pages the national ideas of therapeutics as they are applied to-day by the best practitioners, and as they are likely to remain, with a few modifications, for some decades yet.

This new revised edition is produced, the author tells us, in order to conform it to the sixth decennial revision of the United States Pharmacopœia, though one soon expected to see a new edition of the Materia Medica, for they have been supplied at the rate of one edition for less than two years since the appearance of the first; a phenomenon rarely seen in medical literature.

The methods of Prof. Bartholow are at the same time scientific and simple. His classification of the subject will be well appreciated by students especially, and the bibliography which appears at the end of each article, is a commendable feature which is found in all the leading scientific books of the day, and facilitates study.

However, in spite of so many commendable arrangements, the work fails to give us the doses of medicines in the metric system, and will thereby help more than others to retard progress in a practical direction. Although its pages are most likely stereotyped, the dose of most drugs is stated in a separate paragraph, and it seems that the cost of adding metrical figures would have been slight.

A late review of this book complains of some discrepancy between it and the new pharmacopœia. We only find a slight difference in regard to laudanum, and it is one which is not likely to lead to any mischief, in our opinion, and the profession at large will be thankful for this new edition.

The enthusiasm of the author for the treatment of disease by the hypodermic syringe has been blamed by some, but we are persuaded that a more scrupulous cleanliness in the physician, and a little less ignorance in the community, will eventually bring that method into even a higher repute and more extensive use than Bartholow recommends now. The daily use of the syringe in our hands has failed, as in hundreds of others physicians' hands, to produce a single abscess yet, but the antiseptic precautions necessary in its use were always observed.

H. D. V.

---

HAND-BOOK OF ELECTRO-THERAPEUTICS. By DR. WILHELM ERB, Professor in the University of Leipzig. Translated by L. PUTZEL, M.D., with 39 wood-cuts. June No. of Wood's Library. 8vo, cloth, pp. xi–366. New York: William Wood & Co. 1883.

The first 32 pages of this volume are occupied by a physical introduction, from which all elementary knowledge of electricity has wisely been omitted, as it is supposed that every physician has some notions of electrical science. Follows a physiological introduction of 25 pages, in which the general effects of electricity on the living body are briefly stated. Then comes Electro-Diagnosis, in which Erb has gained so much reputation, especially in connection with the Reaction of Degeneration. Many brief cases are reported to illustrate the laws of electrical action on different classes of cases. The fourth part of the volume is given to General Electro-Therapeutics, beginning with page 103.

After a few remarks on the most important electro-therapeutical theories, that of Dr. Poole, of Ontario, that electricity is a paralyzer, being omitted, the author tells us that now, as formerly, electro-therapeutics must be studied on an empirical basis. Of the electrical bath Erb says: "*A priori*, it cannot be denied that the electrical bath may produce very marked effects, but the therapeutical experience hitherto obtained is not calculated to inspire great faith in its efficacy." The author believes that the most important principle is the treatment in loco morbi.

Part V, and last, treats of Special Electro-Therapeutics, and occupies the most of the volume. Diseases of the Brain, including the Psychoses; Diseases of the Spinal Cord; Diseases of the Peripheral Nerves; Paralysis and Atrophy; Neuralgia; Spasm; Neuroses; and a few other classes of diseases, form the main divisions of this part.

As electricity has not yet come into general use among ordinary practitioners, this treatise will be found by them amply sufficient.

H. D. V.

---

ELEMENTARY PRINCIPLES OF ELECTRO-THERAPEUTICS. For the Use of Physicians and Students, with 135 Illustrations. Prepared by C. M. HAYNES, M.D. Published by the McIntosh Galvanic and Faradic Battery Co., Chicago, Ill. 8vo. 420 pp. Price, $2.

This is a plain, practical, and complete exposition of the subject of which it treats. It contains suggestions in regard to the selection and care of batteries, comparisons of the different forms of electricity in their physiological effects and therapeutic action, and the application of electricity to diagnosis. It discourses quite in detail on the subject of electrolysis, the galvano-cautery, and the electro-thermol bath. It also contains a full vocabulary of electrical language. The book will be found very useful to the busy practitioner who wishes to get at the practical facts of electro-therapeutics in a clear and succinct form. The illustrations are numerous and many of them original, and add much to the value of the book. The paper, composition, and press work reflect credit on those who had charge of the details of its publication.

On the whole, we can earnestly commend the book to all seeking information upon the very important subjects it elaborates.

D. R. B.

THE MEDICAL STUDENT'S MANUAL OF CHEMISTRY. By R. A. WITTHAUS, A.M., M.D., Professor of Chemistry and Toxicology in the University of Buffalo; Professor of Chemistry and Toxicology in the University of Vermont; Professor of Physiological Chemistry in the University of the City of New York; Chemist to the City of Buffalo, etc., etc. 8vo, cloth, pp. xi–370. New York: William Wood & Co. 1883.

"In venturing to add another to the already long list of chemical text-books, the author trusts that he may find some apology in this, that the work is intended solely for the use of a class of students whose needs in the study of this science are peculiar.

"While the main foundations of chemical science, the philosophy of chemistry, must be taught to and studied by all classes of students alike, the subsequent development of the study in its details, must be moulded to suit the purposes to which the student will subsequently put his knowledge. And particularly in the case of medical students, in our present defective methods of medical teaching, should the subject be confined as closely as may be to the general truths of chemistry and its applications to medical science." (From the Preface).

This is the third Manual of Chemistry written for medical students by the author within a few years, and we do not believe that a more competent writer exists in America on the science therein treated.

The philosophy of the subject is briefly but clearly stated in the first 38 pages, in which are contained remarkably clear definitions of the properties of matter, its general and physical characters, and its chemical laws of combination. The classification of elements here adopted is surely commendable, although we fail to see why, from a philosophical standpoint, hydrogen and oxygen should form a class by themselves.

Organic chemistry, alcohols, ethers, animal fats, urine, etc., is not made a division by itself, but follows the carbon group, and forms the larger part of the volume. This is certainly a more rational treatment, but whether it will be found more useful and practical, time will show.

The few pages of laboratory technics inserted as part III, at the end of the volume, will be of much use to the student, and one would wish that a larger part of the work was given to the methods of forming a laboratory for private study, but the author is content in this that "although the Manual puts forth no claim as a work upon analytical chemistry, we have endeavored to bring that branch of the subject rather into the foreground so far as it is applicable to medical chemistry. H. D. V.

---

SEXUAL IMPOTENCE IN THE MALE. By WILLIAM A. HAMMOND, M.D., Professor of Diseases of the Mind and Nervous System in the New York Post-Graduate Medical School, etc. Small 8vo, cloth, pp. 274. $2.50. New York: Bermingham & Co., 1883.

The fascinating style of Dr. Hammond is recognized in every page of this book, which is also rich in original and unique observations. Never before has the subject of impotence received a more careful nor perhaps a more extensive treatment, and, though the reader's natural conclusions will be sad, and will lead him to look with pity on human miseries; though he may philosophize on the *impotence* of therapeutics as applied to-day; yet, he will profit by this interesting statement of the most degraded phase of human morality. The varied experiences of Dr. Hammond enabled him to exhaust thus his subject, and this and other features will more than make up for the scientific methods in which this monograph is lacking. The four chapters into which the book is divided are the following: Absence of Sexual Desire; Absence of the Power of Erection and of Consequent Intromission; Absence of the Power of Ejaculating the Seminal Fluid into the Vagina; Absence of the Ability to Experience Pleasure during the Act of Copulation and during the Emission of the Semen.

This book, we are sorry to say, will offer quacks a rich source of references, and help them more than any work to terrorize and victimize the class on which they thrive. H. D. V.

